# Supplementary material for: Patterns of Intron Gain and Loss in Fungi
Source: PLoS Biol. 2004 Nov 30;2(12):e422. doi: 10.1371/journal.pbio.0020422 (PMC532390; doi:10.1371/journal.pbio.0020422)
Supplement: Table S1 — Also available at http://genes.mit.edu/NielsenEtAl/. (4.3 MB ZIP). [file pbio.0020422.st001.zip › NielsenEtAl/html/294.html]

AN4349.1.NCU01659.1.MG01106.1.FG06284.1


```
 CLUSTAL W (1.82) Multiple Sequence Alignments - Introns Inserted


Sequence 1: NCU01659.1	1226 aa
Sequence 2: MG01106.1	1136 aa
Sequence 3: FG06284.1	1151 aa
Sequence 4: AN4349.1	1125 aa
Alignment Length: 1268 aa
Number Identitical Residues: 504 aa
Alignment Score (without introns) 25949


MG01106.1 	----------MDNSSTPLADYFWIAGIENISYGDS----SLPS--PNFDQTITEDGEGES
NCU01659.1	----------MDNSSTPLADYFWIAGIENITYDDDRQEESLGASLDTVEDTIIEDGEGET
FG06284.1 	----------MESSSTPLAEYFWIAGVESVSYQDP---NSQPAPAVPVESTIIEDGEPED
AN4349.1  	MPFPTSSSAAETTSASPLADYFWIAGVDGSEILDT---FRKLGDEYRIASSTTAPGPAVT
          	 . .::::::  .*::***:******::.    *        .    . .:    *    

MG01106.1 	ASKE--KDAR--STPTAS--ARHSRHNSA-SRFSKVSQDGTFHLNFSHLEDDGNTLSNRS
NCU01659.1	EGEDELGDASGISSPRAGGHPRHSRQNSG-NRLSALSKLSYNPSLSSDNDIEGNTRSNRS
FG06284.1 	EWTNG-------GQPKTN--ARHSRQNSA-SRLSKMSLTERFSIQTLD-DTDGNTKSNRS
AN4349.1  	DIIEEDADAEDAHDRFDGLLSRSSSVIGPRGSFQRLSFRSEDADQNANGNANG-THSNRS
          	   :   .: .      .  .* *   .  . :. :*          . : :* * ****

MG01106.1 	SATIRLVPP---------------------------------VN-------SSASQNGNP
NCU01659.1	SATIRPLPLPNLGNGTGNGNSNNASSGNATNANNTNGAGANGVNGTNGNNKTSPAASPNP
FG06284.1 	SATIRAVNPPDFSNG----------------------------NDDSNGNSQNPAPSGTL
AN4349.1  	SMTIK----------------------------------------------GGGNGSAAS
          	* **:                                               .   .   

MG01106.1 	NGVPEFD----------------FDKALLKFASEREVFLDDLTFSAGAKLQTRPPMVNPR
NCU01659.1	NQIPMFDALGQIPIQDGFLSDLDFDKALLKFAAERENFLDDLTFSAGAKLQSRPPMVNPR
FG06284.1 	GEGSMFMG------------DFDFDKALVKFAAEREVFLEDLTFSAGAKVQARAPMVNPR
AN4349.1  	PRASQFLG----------LEEFDFDKALVKFASERETFLSDLSLSAGAITPASRPRSRLR
          	   . * .           .. .*****:***:*** **.**::****   :  *  . *

MG01106.1 	TERIKADEAESGRKSPLKSIKGSIRRKISFRDMNSMRRQPTAPRP-----AS1TRQT---
NCU01659.1	TEKLRVDDDQSGRKSPLKSIRGSIRRKISFRDMSSARKQPMTPRAGTKPTES~TPRVIPN
FG06284.1 	MERIRAEESDSGRLSPLRSIKGSIRRKMSFRDMNSVRKQPSNRIS-------~TSRA---
AN4349.1  	TQKIVSEEPQ--QSGLLRSSIGSVRRHMSFRDMNSMKRQPSVARQ-------~-------
          	 :::  :: :  : . *:*  **:**::*****.* ::**                    

MG01106.1 	-----------AA~SIRTAKRLSNYNSVIPPPEPLNTDPNMHPLKRRFEPTLLDRYPPRD
NCU01659.1	PYRAYTHPPLPPA~SIRTTKRLSNYNSVIPPPEPLNADPDMHPLKRRFEPVLLDRYPSRD
FG06284.1 	------------A1SIRTTRRLSNYNSVIPPPEPLNTDPDMHPLKRRFEPVLLDRYPPQE
AN4349.1  	------------A1SIRTSRRLSNYNSVIPAPQPLEISPTMHPLKRRFEPVLLDRYPPKG
          	            * ****::**********.*:**: .* **********.******.: 

MG01106.1 	AS-DELARRGRFPDYVPMFAFPNDIQIVSSDDRPRSTWHGFTMTSEDGSKIYGVAVTVWT
NCU01659.1	ATVDELSRRGRFPDYVPMFAFPNDIQIVSSDDRPRSTWHGFTMTSDDNSKIYGICIIVWT
FG06284.1 	AG-DEISRRGRFPDYVPMFAFPNDIQIVSSDDRPRSTWHGFTMTSDDNSKLYGITIIIWI
AN4349.1  	MA-EDLKQRANFPDYVPMFAFPNDINIVSSDQRPRSTWHGFVMTSDNGSRLHAVCVIVWI
          	   ::: :*..**************:*****:*********.***::.*:::.: : :* 

MG01106.1 	ALKADVAEEIEQKCEQWRQSHMSNEERELAASLGVRLAAERANLSQLLAQLPLVASGSVA
NCU01659.1	ALDAETADKVEKRCEQWRQRHMSNEERELAASLGIRLAAERAHLSQLLSELPSIPSGSAA
FG06284.1 	ALNADVAEDVEKKCEEWRQSHMSEEERELAASLGVRLAGERTHLSQLLAKLPTIPSGSPA
AN4349.1  	PLNHEAADQLEKCCEEWRKDNMTDEERELAASLGERLAQERAKLSRLLAQLPTVPSGSES
          	.*. :.*:.:*: **:**: :*::********** *** **::**:**::** :.*** :

MG01106.1 	RESLDDQISSVEEKIGLMTEMLRPLRHGAASKIDGLTAGESGLWSPRAYGIIGSDASKVS
NCU01659.1	RESLDEQISSVEEKISLMTEMLRPLRHGAASKIDGLTAGETGLWVPRAYGILGRDPSKMT
FG06284.1 	RERLEDEISTVEEKITLMTDMLRPLRHGAASKIEGLTAGESGLWAPRAYGILGRDAGNMS
AN4349.1  	REQLEDEISAVEEKIGLMTDLLRPVRHGAASKIEGLTDGDTGFWIPRAYGILGREENMTS
          	** *:::**:***** ***::***:********:*** *::*:* ******:* : .  :

MG01106.1 	FWKEWLRAIVVPMTDGAVLRVPPSSPRVGRWQPLERYVVNLCTEAFSPISSKTQVELGVR
NCU01659.1	FWKEWLRAVTVPMTDGAILRVPPSSPKIGRWQPLERYVVNLCTEAFSPLSSLTQVELGVR
FG06284.1 	FWKEWLKAIVTPMTDGSVLRIPPSSPRVGRWQPLERYVVNLCTEAFNPLGSKTQVELGVR
AN4349.1  	FWKEWLKAVVVPMTDNSVQRVPPASPRMGSWQPLERYVMNLCTEAFAPNTSKTQVELAVR
          	******:*:..****.:: *:**:**::* ********:******* *  * *****.**

MG01106.1 	ELRLYARNEADNELPGSRSTDLYALFRCLSLDNIVALFEYAMSESRIIFVSQHTAMLHLA
NCU01659.1	ELRLYARKEAANELPGSRNIDIYALFRCLSLENIVLLFEYAMSESRIIFLSSHTGMLHLA
FG06284.1 	ELRLYARKEADNEIPGSRTIDIYALFRCLSLENIVALFEYAMAESRIIFLSSHASMLHLA
AN4349.1  	ELRLFARKEATNELPGSRNTDLYALFRTLSLANIIILFEYALTESRIIFLSSHTSMLYLA
          	****:**:** **:****. *:***** *** **: *****::******:*.*:.**:**

MG01106.1 	CHALVSLLYPLKWASVFIPVLPARLLQALDAPCPYIVGIEKRYENIQLPDDDYVLVDLDK
NCU01659.1	CHALANLLYPLKWASIFIPVLPARLISALEAPCPYIVGIERRYENIQLPDDDYVLVDLDK
FG06284.1 	CHALANLLYPLKWASIFIPILPARLLSALEAPCPYIVGIERRYDNIELPEDDYVLVDLDK
AN4349.1  	TRALVDLLFPIPWSGVLIPILPARLIQALEAPCPYIVGIERRYEKVELPSDDFVLVDLDQ
          	 :**..**:*: *:.::**:*****:.**:**********:**::::**.**:******:

MG01106.1 	DTIDATAQPTRLPRQHRRKLFALLQVAAAHRLRYGVEPGPPPYAIEAFPYDSFSVENQSL
NCU01659.1	DIIDATAQAVPLPRQHRRKLMSLLQIAAPHKLRYGVATGPPPYAIEAFPYDAFSAENEAL
FG06284.1 	DTIDATSQPVRLPRQARRKLMSLLQVAAPHKLRYGVATGPPPYAMESFPYDAFSTENGSL
AN4349.1  	DIIESTVKPTPLPRHQRRKLHSLLQLAAPHHSRYGVPVGPPAYAIETFPFDAFMSENSSI
          	* *::* :.. ***: **** :***:**.*: ****  ***.**:*:**:*:*  ** ::

MG01106.1 	FTSTPPKSSLGRWVSQNSATFGEPDLSNPPQPP-LFNAFLRSKHE--------QGRSERP
NCU01659.1	FNPSAKASTLGKWVTQNSSTFAEPDPPNSMRTP-LFNAFSQARPD--------SGHG-RP
FG06284.1 	FRTSTPKSTLGKWVSQSSSGFGEADPPNEIQPP-IFNAFASAHVE--------NGKSDRP
AN4349.1  	YTSKAQSTQLSKYVSLNSNAFGQGSVSLTSHPPLIFNAFLHARNEQTASRGYSSKSSDRP
          	: ...  : *.::*: .*  *.: . .   :.* :****  :: :.::: . :.  ..**

MG01106.1 	TTPMSTR-TSPPAS--LSPISMNFPPMPTTPVSRSDSGFAAGPTTLREKRSTHFDD-RSR
NCU01659.1	TTSKSGK-GSPPSS--VSPVSMHFPPAPTTPVSRSDSGFAVA-TTLREKRSGHFDE-KSR
FG06284.1 	STSKSGK-TSPQSS--VSPVSINFPPMPSTPVSRSDSGFALA-STLREKRSGHFGEEKMR
AN4349.1  	GTGSTFKPGSPPSPRDSSPISGHFPPPPATP--RNDSGMALQ-ASLREKRSGHFDA-ASR
          	 *  : :. ** :. . **:* :*** *:**  *.***:*   ::****** **.    *

MG01106.1 	RSSSYGLD--GRNGGVLHKPSLPVLNGGHNPSLSVSAISIDSQS-----SYGGPGIGYAP
NCU01659.1	RSSSFGMDKNGPPHPPLHRPSLPFLIG-HAQNLSISSISIDSQS-----SFG----AYAP
FG06284.1 	RSSSFGVD----KHPPFQKPGMPFLNG-HQANLSISAISVESQNSVIGGANGGYGGGYAP
AN4349.1  	RSSSFGMD----MRQVPRRPSAPFLG--HAPNLSVSTLNTDFQS------------TYAP
          	****:*:*         ::*. *.*   *  .**:*::. : *.             ***

MG01106.1 	STYAQSTIAASTVMPQMLVQPVTSTDTTVWVEGHCFNWTPRASTSTMCDVCDERCESDDT
NCU01659.1	STYAQSTIAASTIMPNMMVQPVKNTENTVWVEGHCFNWTP-SEQDSICTICDDRSEGNGL
FG06284.1 	STYAQSTLAASTIMPSMQIQPVRNTETTVWVEGHCFNYMP-KDNTSMCTICNDLAEGDGV
AN4349.1  	SVYAQSTVAASTIVPQPAIQPIHNNDGTSWVEGHCLQLQP-WDDKAVCTVCDERADEN-M
          	*.*****:****::*.  :**: ..: * ******::  *  .  ::* :*:: .: :  

MG01106.1 	FKCAGCGCISHGRCLGVACLVCPIAFDAERVRAAFVRCLASLLYTYRKHLGRPSREQKAN
NCU01659.1	YKCSGCNCLAHNHCLGFASLVCPAAFHPDRVRAAFVRCMASLLYTYRKHLGRPTKEQKAN
FG06284.1 	YRCTGCKIVSHGRCLGYCSLICPEAFHADRVRAAFVRCLASLLYTYRKYLGRPSKQQKSN
AN4349.1  	YKCVSCKTQVHNRCAHQICIVCPAAFYPEQVRAAFVRCFASLFYTYKKYLKPASGDKKKA
          	::* .*    *.:*    .::** ** .::********:***:***:*:*  .: ::*  

MG01106.1 	GQLYAFDMAGFIRSLPYDQQDYAVMMRDTQA1FNEFVHERERS--SPSNPQIRLFDEILL
NCU01659.1	GQHYAFDMDGFIRSLPYDQQDYATMLRDTQA1FNEFVHERERI--PPSDPAIRLFDEVIL
FG06284.1 	GQLYAFDMDGFIKSLPHDQHDYAAMMRETQC1FNEFIHDREMQ--PANDASIRLFDEIIM
AN4349.1  	GLTYSFDMDAFCKSLPSEHAEYIGVLSQTQG1FNEFISDRERVNPKARDPRITLFDEIVL
          	*  *:*** .* :*** :: :*  :: :**  ****: :**  .. . :. * ****:::

MG01106.1 	AKKARGRPTMGT-LSRLSTIRVSHGASLG---SHSTPGRLTKVAGYLGDTSDHIWRTASV
NCU01659.1	AKKARGRPVFSQGLSRLSTIRASHGVSAFG-PSSASAARQNKNPGYLNDTTDHIWRTASV
FG06284.1 	AKKARGRSGLATGLSRLSTLRASHGASTYGGFGPARGGSNSKIPAFLGDISDHIWRTASV
AN4349.1  	SKRNRGR----------SSIFSGR-----------------STTDFLSDTSNHLWRTASA
          	:*: ***          *::  .:                 . . :*.* ::*:*****.

MG01106.1 	P--FPN-AKFPG--DYRSVITRV~PTKLDPTLMKEPRAIQGVPRAEQGGRRGLVRKQVPS
NCU01659.1	P--VPSSTKFNDGDNYRSIVTRV~PARLDPTLMKEPRAIQGVPRPEQGRVRGLIRKQVAS
FG06284.1 	P--LPK-GNFPG--EYKTIVTRT~PARLDRSLMREPRSIQGMPRVEPRGTRGLIRKQVPN
AN4349.1  	ASFAASRPQQSISGDYTRVVSRA1PAKLDTSLMKEPRMIHGAPRVSKTAN-NARRKPLPK
          	.:  ..  :   ..:*  :::*. *::** :**:*** *:* ** .     .  ** :..

MG01106.1 	TLN-ATAASPV-
NCU01659.1	MLGPPTPDFRVP
FG06284.1 	MFGTTPPT----
AN4349.1  	LMNGLAISPP--
          	 :.  .
```
